# Enhancement of thermal energy transfer behind a double consecutive expansion utilizing a variable magnetic field

**DOI:** 10.1038/s41598-024-60953-3

**Published:** 2024-05-03

**Authors:** Hamid-Reza Bahrami, Mahziyar Ghaedi

**Affiliations:** Department of Mechanical Engineering, Qom University of Technology, P.O. Box 37195-1519, Qom, 37181 46645 Iran

**Keywords:** Ferrofluid, Heat transfer, Magnetic field, Backward facing step, Ferrohydrodynamic, Forward-facing step, Sudden expansion, Sudden compression, Mechanical engineering, Chemical engineering

## Abstract

This research focuses on utilizing non-uniform magnetic fields, induced by dipoles, to control and enhance thermal energy transfer in a two-dimensional cooling conduit including a double backward-facing step. The presence of electronic equipment along the straight channel path creates such arrangements, and cooling is often ineffective in the corners of the formed steps. The use of a non-constant magnetic field is a passive technique to improve the cooling rate in these sections without changing the internal geometry, thereby increasing the heat transfer rate. A commercial software based on the finite volume technique is employed to solve the governing equations of fluid flow and heat transfer. Multiple parameters are examined in this study, including the flow Reynolds number (12.5–50), dipole location and strength (0.1–5 A-m), and the number of dipoles (single or double). The results indicate that all of these parameters have a significant impact on the thermal energy transfer. The results of the study show that a single dipole increase the average heat transfer by about 22%, two magnetic fields by 40%, the strength of the magnetic source by 24% with respect to the non-magnetic field in the present study.

## Introduction

The primary purpose of mini-channels design is to facilitate the cooling of multiple elements in their path. Consequently, modifications to the channel's cross-section become necessary to accommodate the obstacles requiring cooling. However, these alterations have implications for heat transfer efficiency, as they introduce sudden changes that give rise to undesirable phenomena, such as flow disruptions or wakes, which hinder the heat transfer process. These abrupt modifications are commonly referred to as sudden expansions or sudden compressions (or backward/forward facing step) in the literature. The nature of these changes can involve either a single or multiple compressions or expansions. The undesirable phenomena are more complicated in multiple ones.

Researchers have proposed several solutions to address these limitations^1–3^. For instance, Talaei and Bahrami^4^ implemented various techniques, including corrugation and the use of porous/solid baffles, to enhance thermal energy transfer in the region after a single backward-facing step. Their findings indicate the existence of an optimal location for the porous baffle on the opposing wall of the step. Bahrami^5^ conducted a research to explore the influence of a semi-porous baffle on thermal energy transfer enhancement after a two-dimensional sudden expansion. The research findings highlight the significance of the relative length of the porous section. It was observed that an appropriate adjustment of this length led to a substantial improvement of 90% in thermal energy transfer compared to the case without any manipulation. Nath and Krishnan^6^ have optimized heat and mass transfer after a two dimensional sudden expansion using the Taguchi technique and utility concept. Significant parameters are identified, and the utility concept provides slightly lower Nusselt and Sherwood numbers compared to the Taguchi method. Selimefendigil and Öztop^7^ employed a combination of a rotating cylinder and a uniform magnetic field to augment thermal energy transfer behind a step. Their findings revealed that utilizing the rotating cylinder alone resulted in a remarkable 244% improvement in thermal energy transfer. Rana et al.^8^ considered the application of various nanofluids to improve thermal energy transfer behind a double step. Their findings demonstrate that, in terms of heat transfer performance, SiO_2_ nanofluid exhibits the highest performance, followed by Al_2_O_3_ and ZnO, when compared to the base case. Different studies on the use of hybrid nanofluids have been conducted by researchers^9–12^. For example, Mohankumar and Prakash^13^ conducted a study utilizing elliptic obstacles to enhance heat transfer behind double backward-facing steps. The outcomes highlighted that the vertical positioning of the obstacles played a crucial role in significantly improving the thermal energy transfer.

Ferrofluids have garnered considerable attention in thermal management owing to their exceptional properties. These fluids can be simply manipulated by an external magnetic field, making them promising for several applications in heat transfer and thermal control. One of the advantages of ferrofluids in thermal management is that they eliminate the need for complex channel geometry manipulation, which can incur additional expenses. This is particularly beneficial in the case of mini channels where the use of micro-electromechanical systems (MEMS) might be required. The external magnetic field required to manipulate the ferrofluid can be generated using a wire or a combination of wires carrying electric current. This allows for precise control over the thermal management process by simply turning the electric current on or off as needed in the channel. This capability provides flexibility and enables efficient and responsive thermal control in various scenarios.

Ferrofluids have also found applications in the thermal management of channels with sudden changes in geometry. For instance, Toumi et al.^14^ employed a fixed finned-cylinder, positioned after a step, along with a uniform inclined magnetic field to enhance heat transfer. Their research indicated that rising the Hartmann number resulted in a deterioration in thermal energy transfer, while the concentration of ferrofluid had a positive effect on heat transfer performance. Manh et al.^15^ employed a variable magnetic field to augment heat transfer following a sudden expansion. Their findings revealed that the strategic implementation of the magnetic field in conjunction with the expansion led to a remarkable 300% improvement in heat transfer. Geridönmez and Öztop^16^ conducted a study on improving thermal energy transfer after a backward-facing step utilizing a hybrid-nanofluid and a partial magnetic field. This research demonstrated that the concentration of magnetic nanoparticles had a significant influence on thermal energy transfer performance. Song et al.^17^ have explored electrokinetic instability in microfluidic systems using ferrofluid/water co-flows. Their results show that instability waves occur at the interface due to the electric field Atashafrooz et al.^18^ examined the flow pattern and entropy generation within a channel featuring an abrupt contraction considering the effect of a magnetic field. Their findings showed that the Hartmann number had a detrimental impact on heat transfer. There are many related research that could be found in the literature^19–26^.

The literature review shows that he increasing complexity of computer systems necessitates smaller, faster electronic equipment, intensifying heat generation and impairing system efficiency. Addressing this, researchers focus on fluid mechanics to develop compact, portable designs. Ferrofluids and magnetic fields offer promising thermal management solutions, especially in cooling electronic devices. However, gaps persist in applying these methods to double backward-facing steps. This study investigates the potential of single or multiple dipoles in such scenarios, employing commercial software to analyze their impact on heat transfer parameters. This configuration presents a more complex scenario compared to a single step. Therefore, this study aims to investigate the potential of utilizing single or multiple dipoles in a channel containing a double backward sudden expansion. A commercial software is employed for the investigation, and the influences of various parameters such as the Reynolds number and strength of magnetic dipoles, flow Reynolds number, and location of the magnetic source on heat transfer are explored.

## Problem description

**Figure 1:**
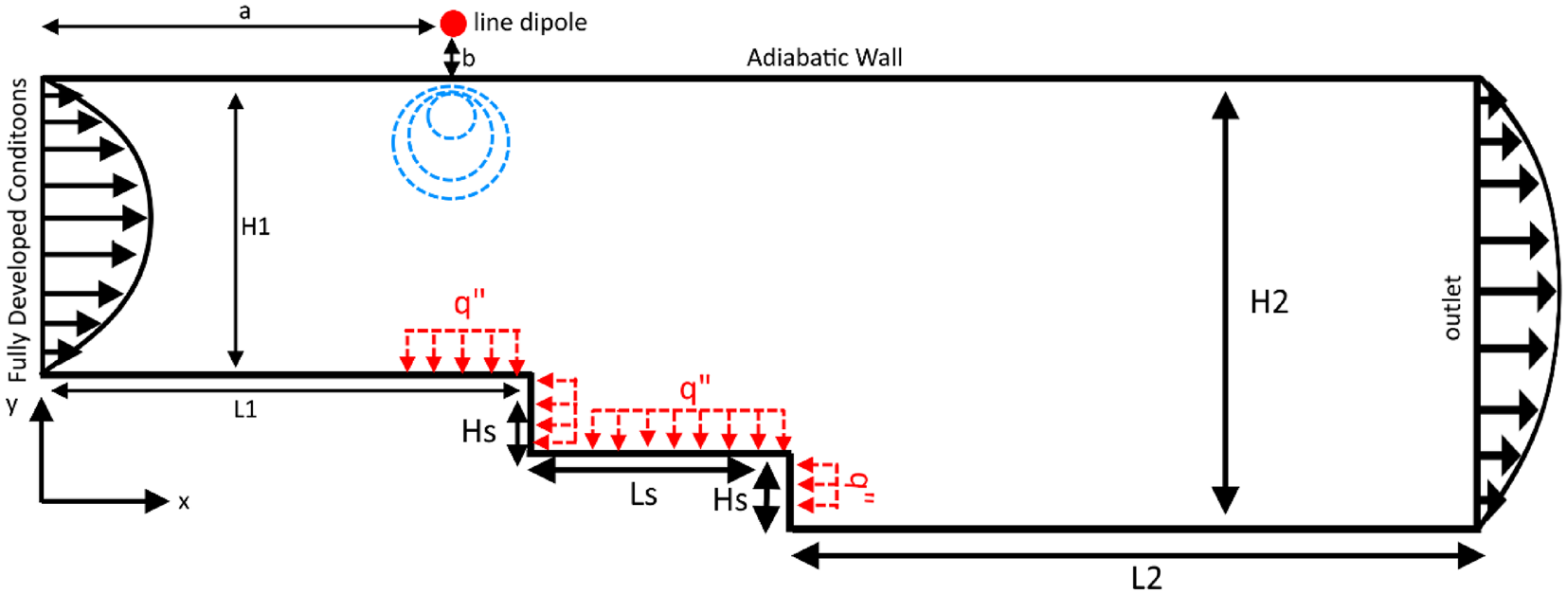
The schematic of the problem under investigation.

It is assumed that a ferrofluid with magnetic properties flows inside a milli-channel featuring a double backward-facing step. The schematic of the channel's geometry, the location of the dipoles, and the boundary conditions are depicted in Fig. 1. The dimensions of the studied channel and the presented in Table 1. The ferrofluid enters the channel with a height of 1 mm (H_1_ = 1 mm) under laminar flow regime. Due to the presence of two sudden expansions, the channel height increases twice, and eventually, the flow enters the main channel with a height of 2 mm (H_2_ = 2 mm). The height of each step is 0.5 mm (H_s_ = 0.5 mm). The length of the channel before reaching the first backward-facing step is 20 mm (L_1_ = 20 mm), the distance between consecutive steps is 1 mm (L_s_ = 1 mm), and the length of the channel from the second step to the channel outlet is 29 mm (L_2_ = 29 mm). All walls are adiabatic, and only the walls of the steps and part of the wall the steps adjacent to the heat source element that have a constant heat flux of 10^4^ W/m^2^, which is chosen based on the literature^27–30^. The flow enters the domain with a temperature of 293 K and hydrodynamically fully developed conditions.

**Table 1:** Dimensions of the geometry (in mm).

Parameter	Value
H_1_	1.0
H_2_	2.0
H_s_	0.5
L_1_	20.0
L_s_	1.0
L_2_	29.0
a	According to conditions
b	According to conditions

### Thermophysical properties

In this study, two commercially available ferrofluids^31^, namely EMG-805 and EMG-308, were utilized to investigate their the impact of their thermal and magnetic properties. The magnetic and thermophysical properties of the working fluids are given in Table 2.

**Table 2:** Thermophysical properties of ferrofluids**.**

Parameter	Unit	EMG-308	EMG-805
Density (ρ)	kg/m^3^	1060	1190
Thermal Conductivity (k)	W/m–K	0.63	0.67
Specific Heat (Cp)	J/kg-K	3915.9	3475.2
Solid Volume Fraction (ϕ)	%	1.2	3.6
Viscosity (µ)	Pa·s	0.002	0.003
Magnetic Sensitivity (*x*_ff_)	–	0.50	2.89
Saturation Magnetization (M_s_)	mT	6.6	22

### Governing equations

This study considers a two-dimensional, steady state, homogeneous, incompressible, single phase, fully developed inlet velocity, and laminar flow regime. It neglects the influences of gravitational body forces and viscous heat dissipation throughout the calculations because the Richardson number is much less than unity. Based on these assumptions, the momentum and continuity equations in the x and y directions, and energy equation including the addition of magnetic dipole as a volumetric force term in the momentum equations. The model, which is used in the current study, is based on the previous research^32–36^ and is explained in the following:

Continuity^37^:

1 $$\frac{\partial {\text{u}}}{\partial {\text{x}}}+\frac{\partial {\text{v}}}{\partial {\text{y}}}=0.$$

Energy^38^:

2 $$\uprho {{\text{C}}}_{{\text{p}}}\left({\text{u}}\frac{\partial {\text{T}}}{\partial {\text{x}}}+{\text{v}}\frac{\partial {\text{T}}}{\partial {\text{y}}}\right)={\text{k}}\left(\frac{{\partial }^{2}{\text{T}}}{\partial {{\text{x}}}^{2}}+\frac{{\partial }^{2}{\text{T}}}{\partial {{\text{y}}}^{2}}\right).$$

x-momentum^39^:

3 $$\uprho \left({\text{u}}\frac{\partial {\text{u}}}{\partial {\text{x}}}+{\text{v}}\frac{\partial {\text{u}}}{\partial {\text{y}}}\right)=-\frac{\partial {\text{P}}}{\partial {\text{x}}}+\upmu \left(\frac{{\partial }^{2}{\text{u}}}{\partial {{\text{x}}}^{2}}+\frac{{\partial }^{2}{\text{u}}}{\partial {{\text{y}}}^{2}}\right)+{{\text{S}}}_{{\text{x}}}.$$

y-momentum^39^:

4 $$\uprho \left({\text{u}}\frac{\partial {\text{v}}}{\partial {\text{x}}}+{\text{v}}\frac{\partial {\text{v}}}{\partial {\text{y}}}\right)=-\frac{\partial {\text{P}}}{\partial {\text{y}}}+\upmu \left(\frac{{\partial }^{2}{\text{v}}}{\partial {{\text{x}}}^{2}}+\frac{{\partial }^{2}{\text{v}}}{\partial {{\text{y}}}^{2}}\right)+{{\text{S}}}_{{\text{y}}}.$$

The components S_x_ and S_y_ represent the volumetric forces generated by the external magnetic field applied to the milli-channel, and the effects of the magnetic field are considered in the momentum Eqs. (3) and (4). To express the magnetic field force in the momentum equations, calculations and equations need to be formulated and simplified in the field of electromagnetics. The laws of electromagnetics and Maxwell's equations are utilized to mathematically express the magnetic field effects. Initially, by applying Ampere's (5) and Gauss's law (6), the magnetic field intensity and magnetic flux are respectively expressed^40^:

5 $$\overrightarrow{\nabla }\times \overrightarrow{\mathbf{H}}=0.$$

6 $$\overrightarrow{\nabla }\cdot \overrightarrow{{\text{B}}}=0.$$

The Langevin model can used to consider the magnetic field. The mentioned model takes into account magnetic saturation in nanoparticles when applying a magnetic field, simulating the conditions and influences of the magnetic source on the ferrofluid flow. The relationship between magnetic field intensity and magnetic flux is mathematically expressed as follows^32^:

7 $$\overrightarrow{\mathbf{B}}={{\varvec{\upmu}}}_{0}\left(\overrightarrow{\mathbf{H}}+\overrightarrow{\mathbf{M}}\right).$$

Next, using the magnetic field vector, the magnetic scalar potential (Eq. 8) of a current-carrying wire is defined. Then, using the mathematical Eqs. (9), the non-uniform magnetic field vector is expressed in polar (10) and Cartesian coordinates (Eqs. 11 and 12)^41^:

8 $$\overrightarrow{{\text{H}}}=-\overrightarrow{\nabla }\cdot {{\text{V}}}_{{\text{m}}}.$$

9 $${{\text{V}}}_{{\text{m}}}\left({\text{x}},{\text{y}}\right)=\frac{\mathrm{msin\theta }}{{\text{r}}}.$$

10 $$\overrightarrow{{\text{H}}}\left({\text{r}},\uptheta \right)=\frac{{\text{m}}}{{{\text{r}}}^{2}}\left({\text{sin}}\left(\uptheta \right){\widehat{{\text{e}}}}_{{\text{r}}}-{\text{cos}}\left(\uptheta \right) {\widehat{{\text{e}}}}_{\uptheta }\right).$$

11 $${\text{r}}={\left({\left({\text{y}}-{\text{b}}\right)}^{2}+{\left({\text{x}}-{\text{a}}\right)}^{2}\right)}^\frac{1}{2}.$$

12 $$\uptheta ={{\text{tan}}}^{-1}\left(\frac{{\text{y}}-{\text{b}}}{{\text{x}}-{\text{a}}}\right).$$

The ferrofluid magnetization (M) can be expressed using the Langevin function^35^:

13 $$\overrightarrow{M}={M}_{s}L\left(\alpha \right)\overrightarrow{H}{\left(\left|\overrightarrow{H}\right|\right)}^{-1}.$$

The maximum achievable magnetization for the ferrofluid can be defined using Eq. (14), where the parameter α represents the magnetic to thermal energy ratio, which can be described using Eq. (15)^42^:

14 $${\text{L}}\left(\mathrm{\alpha }\right)=\frac{1}{{\text{tanh}}\left(\mathrm{\alpha }\right)}-\frac{1}{\mathrm{\alpha }} .$$

15 $$\mathrm{\alpha }=\frac{\uppi }{6}\frac{{\upmu }_{0}{{\text{M}}}_{{\text{d}}}\left|\overrightarrow{{\text{H}}}\right|{{\text{d}}}^{3}}{{{\text{k}}}_{{\text{B}}}{\text{T}}} .$$

In Eq. (15), d represents the average diameter of the solid nanoparticles, M_d_ represents the magnetization magnitude, magnetic vacuum permeability is μ_0_ = 4π × 10^–7^ N/A^2^, and k_B_ = 1.38 × 10^–23^ J/K is the Boltzmann constant. Equation (16) defines magnetization magnitude, and the variable ϕ represent the nanoparticles volume fraction in the ferrofluid. Finally, using Eqs. (5) to (16), the magnetic field influence is expressed as a force per unit volume in Eq. (17) and is considered in the momentum equations^36^:

16 $${{\text{M}}}_{{\text{d}}}=\frac{{{\text{M}}}_{{\text{s}}}}{\upphi } .$$

17 $${{\text{S}}}_{{\text{k}}}={{\text{M}}}_{{\text{s}}}{\text{L}}\left(\mathrm{\alpha }\right)\overrightarrow{\nabla }\left({\left(\overrightarrow{{\text{H}}}\cdot \overrightarrow{{\text{H}}}\right)}^{0.5}\right).$$

Boundary conditions:

The boundary conditions are assumed as follows:

Inlet:

18 $${\text{u}}=6{{\text{U}}}_{{\text{mean}}}\left[\frac{{\text{y}}}{{\text{h}}}-({\frac{{\text{y}}}{{\text{h}}})}^{2}\right].$$

Outlet:

19 $$\frac{\partial u}{\partial x}=0; \frac{\partial T}{\partial x}=0;p={p}_{0}.$$

Adiabatic walls:

20 $$u=0;v=0; \frac{\partial T}{\partial y}=0.$$

Walls with constant heat flux:

21 $$u=0;v=0; k\frac{\partial T}{\partial y}=q".$$

### Necessary relations

The necessary equations which are needed for the problem including the Nusselt and Reynolds numbers, and the hydraulic diameter are expressed as follows:

22 $${\text{Re}}=\frac{\uprho \overline{{\text{V}}}{{\text{D}} }_{{\text{h}}}}{\upmu } .$$

23 $${\text{Nu}}={\text{h}}\times \frac{{{\text{D}}}_{{\text{h}}}}{{\text{k}}} .$$

24 $${{\text{D}}}_{{\text{h}}}=2{{\text{H}}}_{1}.$$

25 $$N{u}_{ave}=\frac{\underset{0}{\overset{L}{\int }}Nu dx}{L}.$$

### Numerical procedure

**Figure 2:**
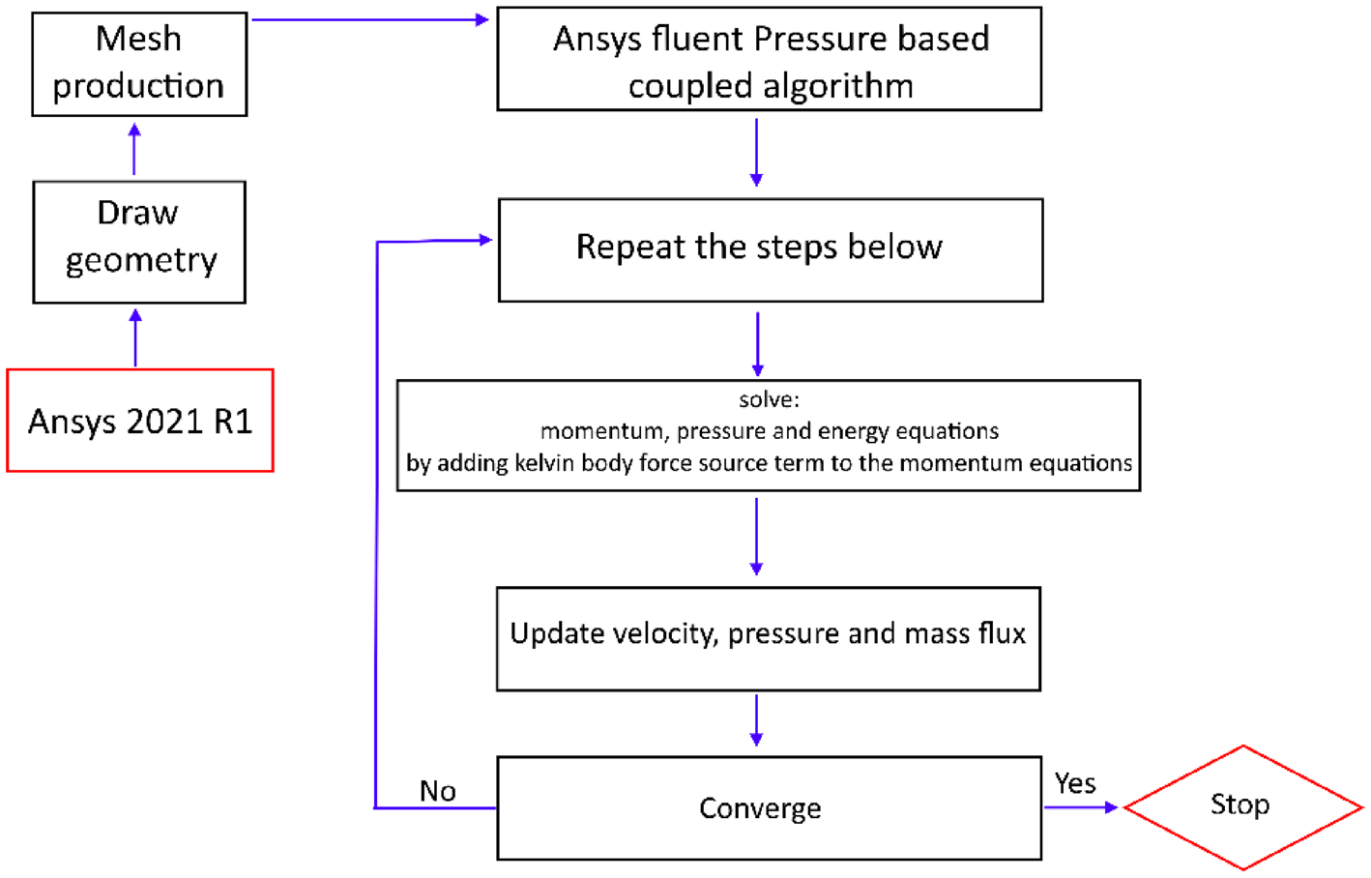
The flowchart of the solution procedure.

For modeling the desired problem, the ANSYS Fluent 2021 software, which is a finite volume-based numerical solver, has been utilized. The solver employs a coupled solution algorithm for pressure and velocity. In this study, in the spatial discretization section, momentum and energy equations are solved using quick and pressure equations using PRESTO! algorithm. For gradient, the least square cell-based technique is applied. To ensure convergence, all equations are solved until the residuals reach a value less than 10^–8^. The modeling is performed using a system with a 7th generation processor running at 2.7 GHz, and the system has a memory capacity of 16 gigabytes. The numerical procedure flowchart is revealed in Fig. 2.

### Verification

**Figure 3:**
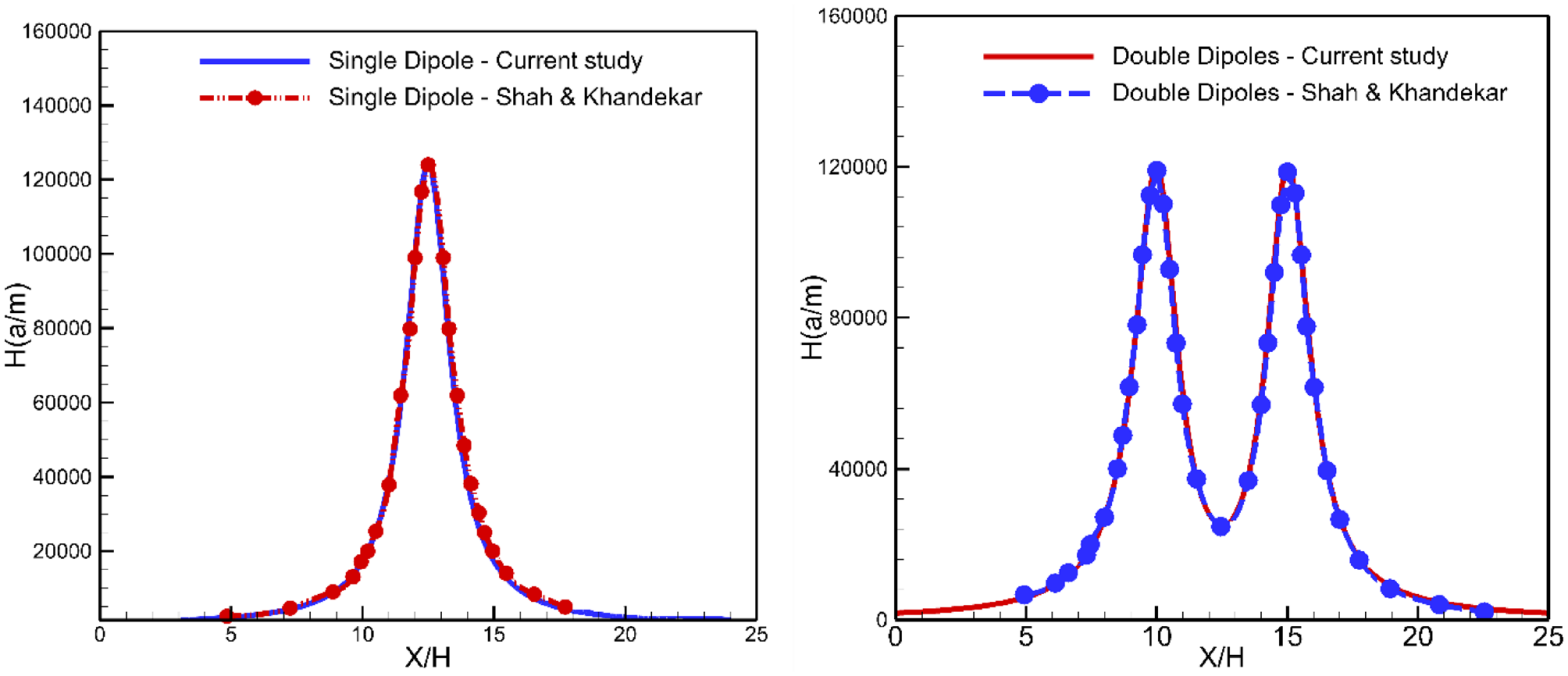
Validation of the magnetic field intensity for single dipole and double dipole with Shah and Khandekar^35^ results.

The accuracy of using the external magnetic field was verified by comparing it with the numerical solution presented by Shah and Khandekar^35^. They investigated the influence of the magnetic field on the laminar flow of the EMG-805 ferrofluid in a two-dimensional conduit. The validation was conducted for a Reynolds number of 25. The comparison of results, shown in Fig. 3, clearly indicates a strong agreement between the applied force on the ferrofluid and the magnitude of the magnetic field. These findings demonstrate a satisfactory level of accuracy in the study.

Other validation cases are also used to verify the current study for reattachment length after a backward-facing step. The study is done with different references and Reynolds numbers. The data and the comparison are given in Table 3. It can be observed that the present research is in suitable agreement with the previous results.

**Table 3:** Comparison of the present research and other published research for recirculation zone length.

	Recirculation length	Error
ER = 1.9423, Re = 10		
Present study	0.54	–
Armaly et al.^43^	0.55	1.81%
Wu and Kumar^44^	0.55	1.81%
Issakhov et al.^45^	0.55	1.81%
ER = 1.9423, Re = 50		
Present Study	1.54	–
Armaly et al.^43^	1.55	0.65%
Wu and Kumar^44^	1.56	1.28%
Issakhov et al.^45^	1.55	0.65%
ER = 1.9423, Re = 100		
Present Study	2.7	–
Armaly et al.^43^	2.65	1.88%
Wu and Kumar^44^	2.63	2.66%
Issakhov et al.^45^	2.63	2.66%
ER = 1.9423, Re = 200		
Present Study	4.6	–
Armaly et al.^43^	4.7	2.12%
Kumar and Vengadesan^28^	4.86	5.34%
Issakhov et al.^45^	4.5	2.22%

### Mesh study

**Figure 4:**
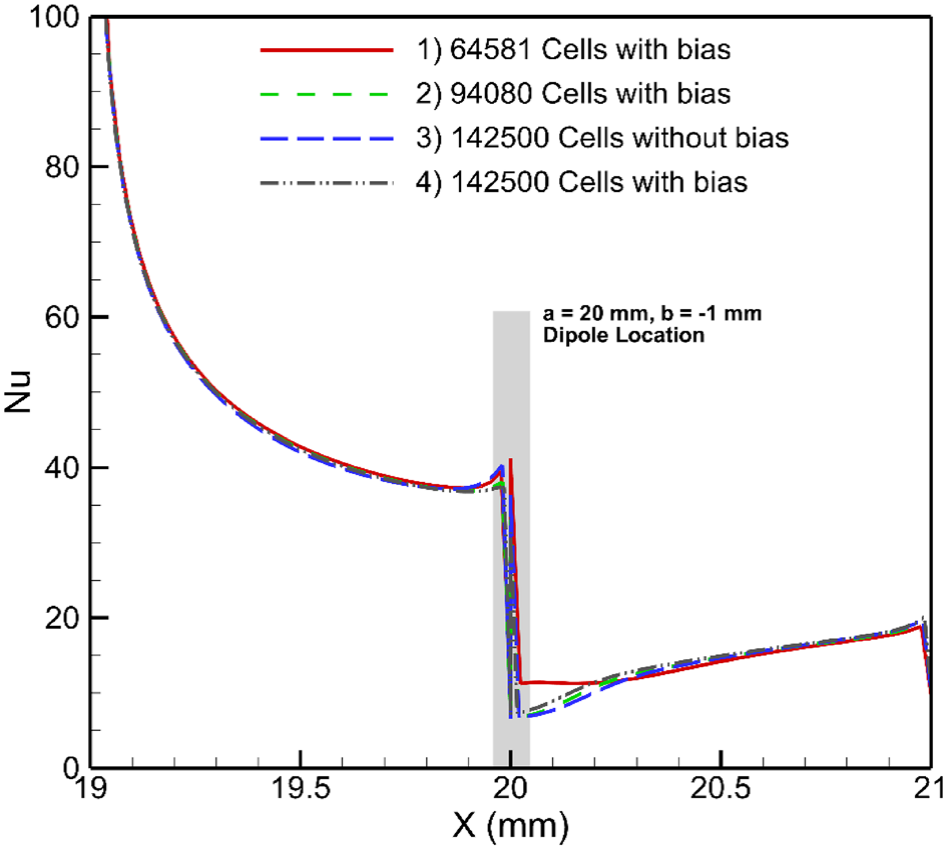
The results for grid independence study (Re = 90, m = 1 A-m, Dipole is placed bellow the lower boundary at a = 20 mm, and b = 1 mm, EMG-805 has used as inlet Fluid).

**Figure 5:**
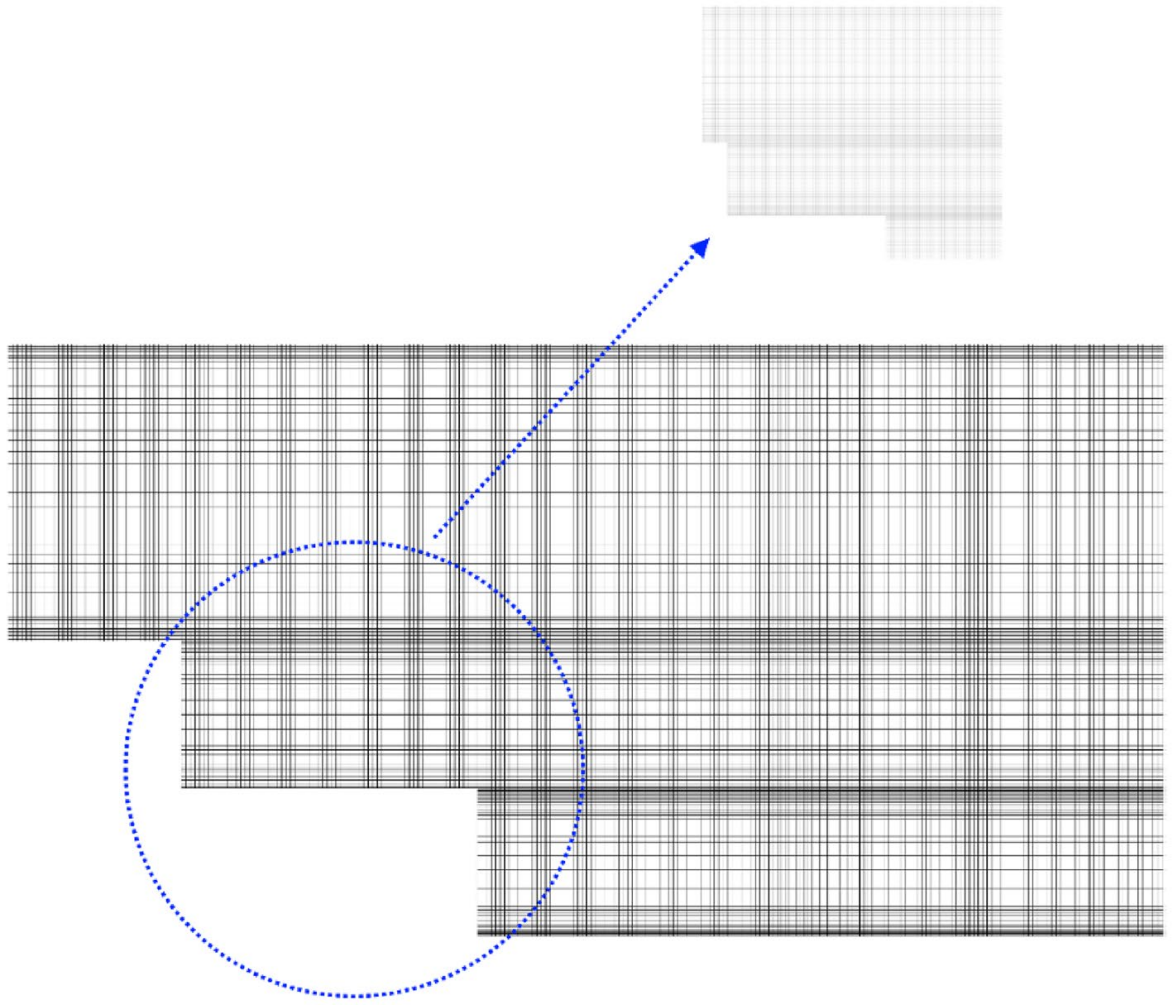
A sample grid.

Various grid numbers were examined for the current problem at base conditions (without employing magnetic fields), and the corresponding outcomes are shown in Fig. 4. Based on the obtained results, grid #3 was selected for subsequent studies. A sample grid generated in this research is also shown in Fig. 5.

## Results and discussion

The purpose of this research is to examine the potential of magnetic dipoles within a channel featuring a double backward-facing step. Numerous factors come into play in this investigation, including the number of dipoles, type of ferrofluid, the flow Reynolds number, and the strength of the dipoles. To develop a comprehensive understanding of the system's behavior and isolate the influence of each parameter, the study initially focuses on examining the impact of individual dipoles on heat transfer. Subsequently, the study delves into investigating the effects of employing two dipoles, as well as varying the Reynolds number and dipole strength. Through systematic variation of these parameters, the objective is to discern their respective contributions to enhancing heat transfer.

### Effects of type of ferrofluid

**Figure 6:**
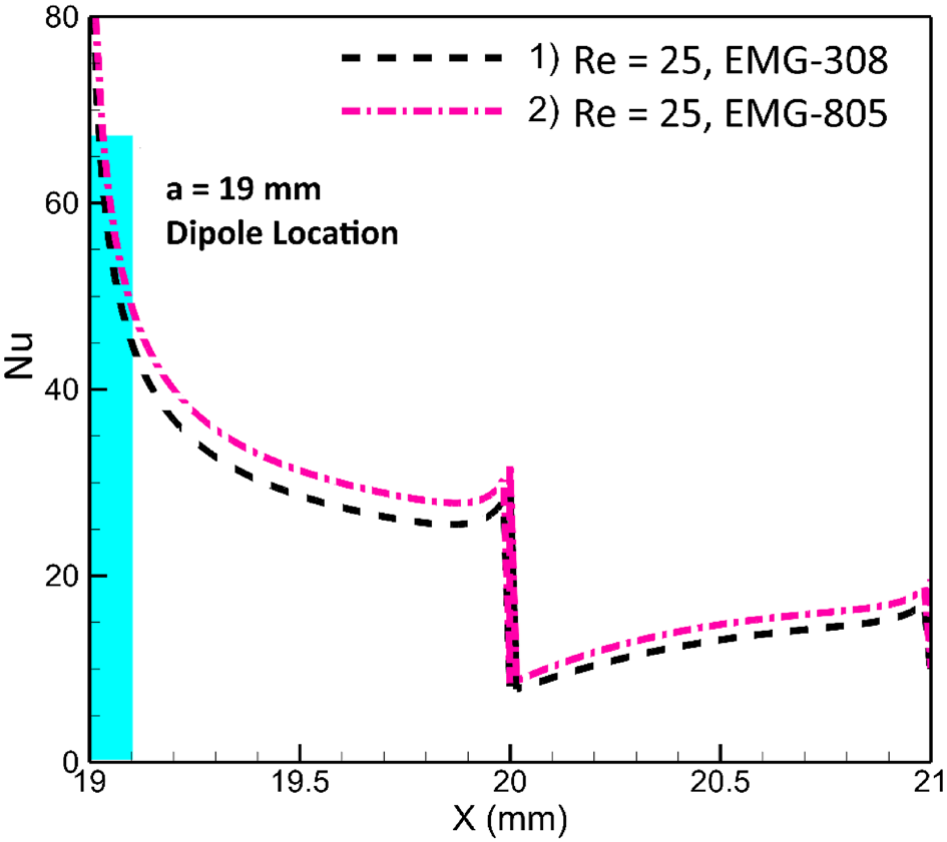
The influences of ferrofluid type on the local heat transfer (m = 1 A-m, Dipole is placed bellow the lower border at a and be 19 mm and 0.5 mm, respectively).

As it has been said before, two kinds of ferrofluids including EMG-805 and EMG-308 have been used in this research. The first has 3.6% and the second has 1.2% volume fraction of magnetic nanoparticles. In fact, the type of ferrofluid in this study shows the volume fraction of magnetic particles. The influences of the nanoparticle volume fraction on the local thermal energy transfer at a fixed location of dipole are shown in Fig. 6. As can be seen, the second ferrofluid, EMG-805, has better performance, showing a better local Nusselt number. The reason for this observation is directly linked to the volume fraction of nanoparticles. EMG-805, containing more Fe_3_O_4_ nanoparticles, exhibits higher thermal conductivity, resulting in better performance. Additionally, it generates a stronger volume force. Therefore, EMG-805 outperforms EMG-308 in terms of local Nusselt number, as revealed in Fig. 6.

### Effects of longitudinal adjustment of a magnetic dipole on the lower border

**Figure 7:**
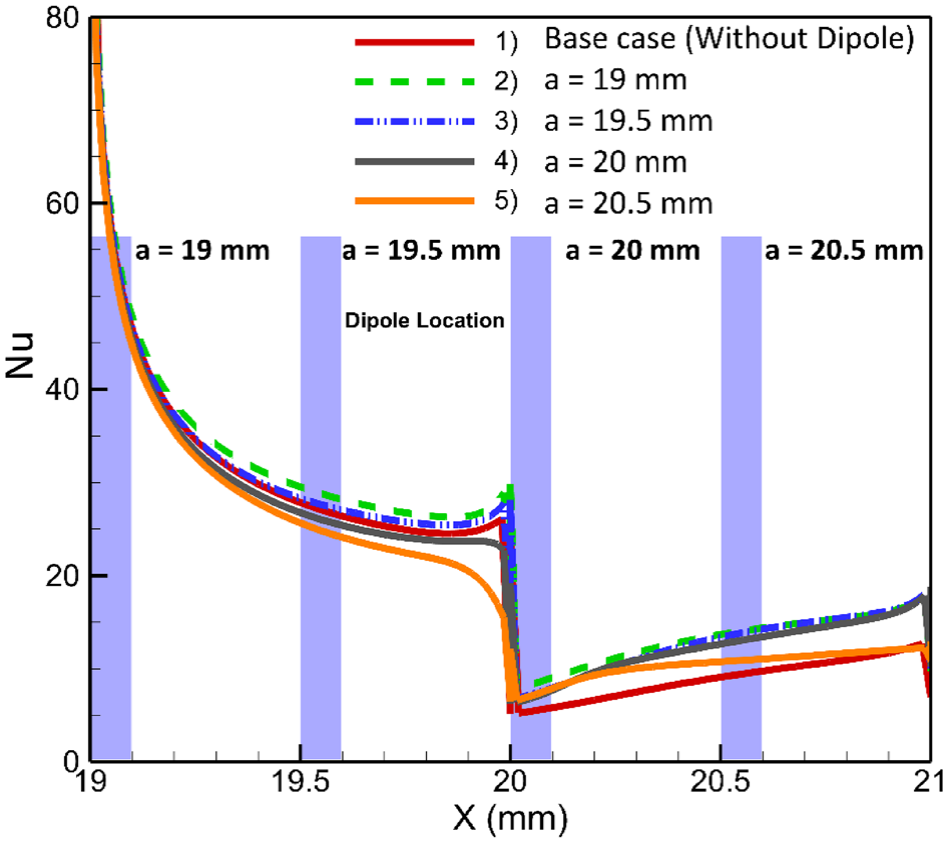
The effects of local longitudinal location of diploes on the local heat transfer (m = 1 A-m, Re = 25, vertical distance of dipole is constant b = 1 mm, EMG-805 has used as inlet Fluid).

**Figure 8:**
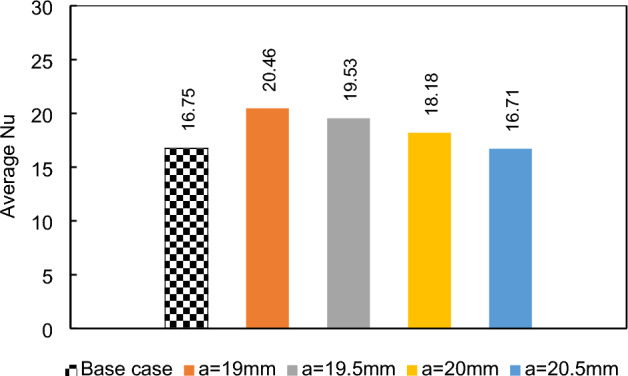
The average Nusselt number for various horizontal positions of the single dipole.

In current section, we will investigate the influence of the dipole's horizontal location on the thermal performance of the system, when it is located bellow the lower border. Initially, the dipole will be positioned at a = 19 mm after the inlet, precisely 1 mm before the step. Subsequently, it will be systematically moved towards the outlet, shifting by 1 mm increments. The local Nusselt number for various conditions is shown in Fig. 7. The presence of dipoles located after the step adversely affects heat transfer compared to the simple case (where no dipole is used). In the case of dipoles located before the step, farer ones give better results. The influences of the magnetic source on heat transfer is attributed to the Kelvin force. The presence of a dipole located behind the step causes perturbations due to the Kelvin force, which in turn enhances heat transfer after the step. This enhancement can be attributed to the mixing that occurs in those locations, while the Kelvin force acts to attenuate forced convection in the main recirculation zone after the steps. To gain a deeper understanding, Fig. 8 provides the average Nusselt number for different conditions. Notably, it is evident that the rightmost case significantly deteriorates heat transfer, even when compared to the simple case. Conversely, the leftmost case demonstrates an improved average Nusselt number, showcasing an enhancement of approximately 22%.

### Effects of vertical adjustment of a magnetic dipole under the lower wall

**Figure 9:**
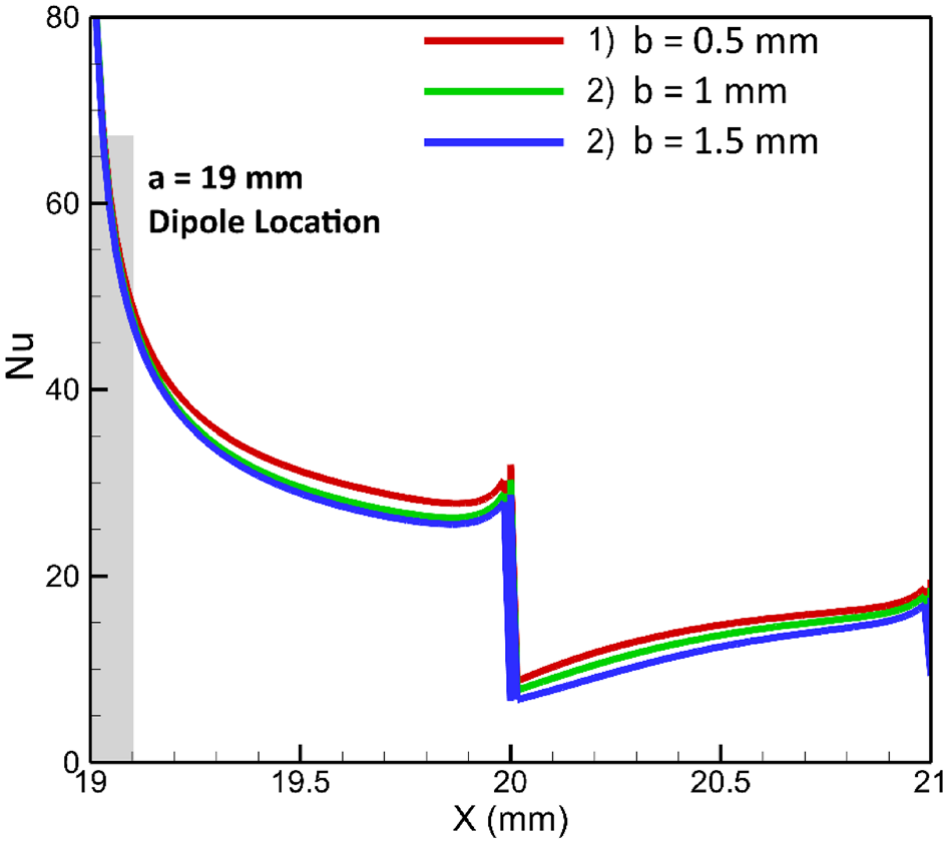
Effects of vertical location of dipole located under the lower border (m = 1 A-m, Re = 25, longitudinal locations of dipole is constant a = 19 mm, EMG-805 has used as inlet Fluid).

**Figure 10:**
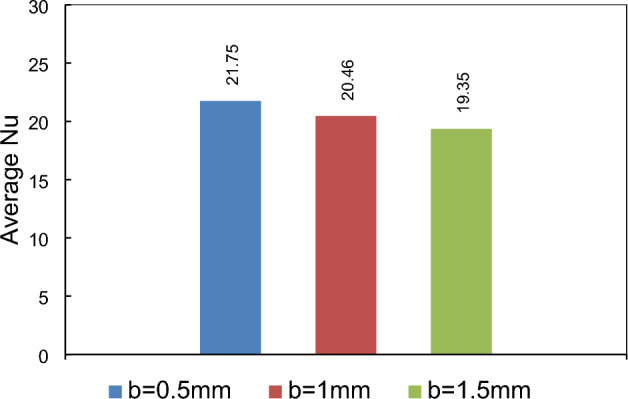
The mean Nusselt number variation in different places of the magnetic source.

In this investigation, we examine the influence of the vertical placement of a single dipole, positioned at a = 19 mm bellow the lower border, on the thermal behavior of the flow within the channel. Three cases are considered, corresponding to b = 0.5, 1, and 1.5 mm. As shown in Fig. 9, it is obvious that as the dipole approaches both the flow and the lower border of the conduit, its impact on the flow becomes increasingly significant. The underlying physical interpretation of this phenomenon is that, in this particular location, the dipole exerts a clockwise rotational force on the flow, effectively attaching flow to the steps’ wall. This attachment aids in heat transfer. As the dipole nears the wall, the magnetic force intensifies, further enhancing flow attachment. Consequently, the undesirable flow detachment diminishes, resulting in improvement in heat transfer. To gain a more comprehensive understanding of the scenario, the average Nusselt number for various conditions presented in Fig. 10 can be considered. Notably, as the dipole approaches the wall within a range of 1.5 mm to 0.5 mm, a notable enhancement in heat transfer of approximately 12% is observed. It is crucial to note that the proximity of the dipole to the wall is contingent upon the wire diameter used in the practical application. In fact, the choice of wire diameter determines the feasible range of dipole vertical placement.

### Effects of longitudinal adjustment of a magnetic dipole on the upper border

**Figure 11:**
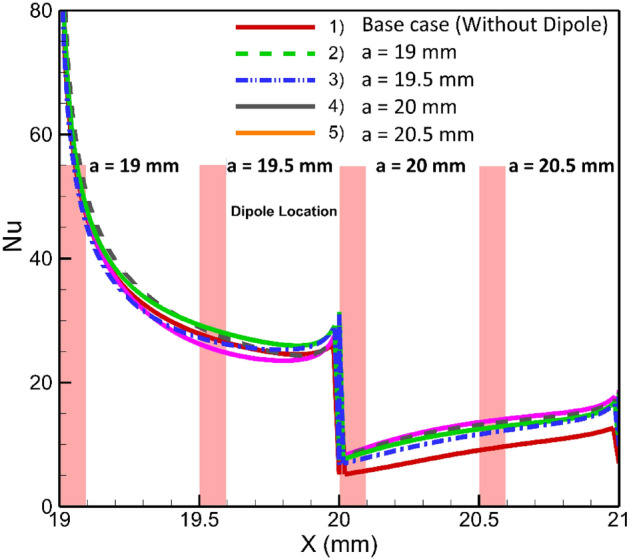
The effects of horizontal place of the dipole located on the upper border on the average Nusselt number (m = 1 A-m, Re = 25, vertical distance of dipole is constant b = 0.5 mm, EMG-805 has used as inlet Fluid).

**Figure 12:**
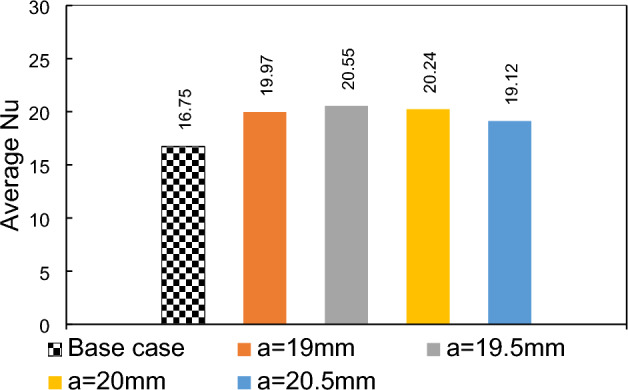
The influences of horizontal position of the dipole located on the higher border on the average Nusselt number.

In the current section, an alternative location for the placement of the dipole, over the upper wall, and different longitudinal positions are investigated. The influence of longitudinal location on the average and local Nusselt number are illustrated in Figs. 11 and 12. These figures indicate that there exists an optimal position for the dipoles, precisely at a = 19.5 mm, which is situated 0.5 mm prior to the steps. At this location, the average Nusselt number reaches 20, exhibiting a 22% improvement. As previously mentioned, the behavior of the fluid in the critical region, specifically over the steps, involves a complex interplay of pressure, viscosity, and magnetic forces. The magnetic force can either exert a positive or negative influence, depending on its placement. This study reveals that the critical point for dipole location lies at a = 19.5 mm; beyond this point, the magnetic field amplifies flow separation, leading to a deterioration in heat transfer.

### Effects of using two dipoles

**Figure 13:**
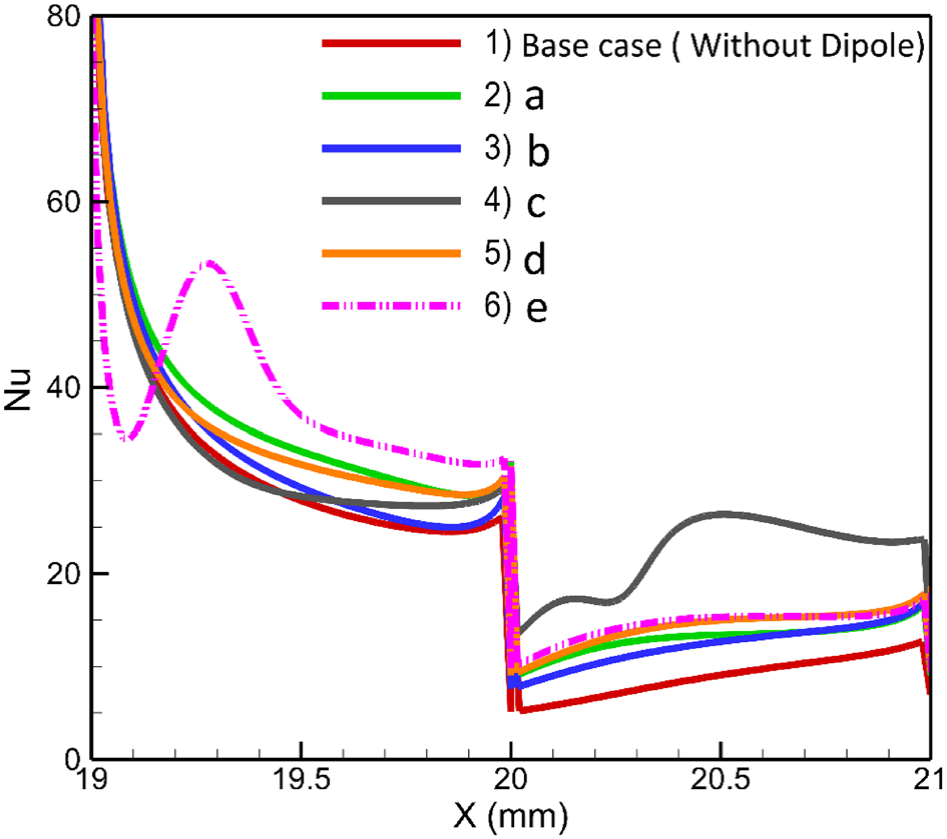
The local Nusselt number variation in different arrangements of double magnetic fields in the domain (m = 1 A-m, Re = 25, EMG-805 has used as inlet Fluid, (**a**) a_1,2_ = 19, 20 mm and b_1,2_ = − 0.5, + 0.5 mm, (**b**) a_1,2_ = 19.5, 20 mm and b_1,2_ =  + 0.5, + 0.5 mm, (**c**) a_1,2_ = 19.5, 20.5 mm and b_1,2_ = − 0.5, − 1.5 mm, (**d**) a_1,2_ = 20, 19.25 mm and b_1,2_ =  + 0.5, − 0.5 mm (**e**) a_1,2_ = 20, 19.25 mm and b_1,2_ =  + 0.5, − 0.25 mm).

**Figure 14:**
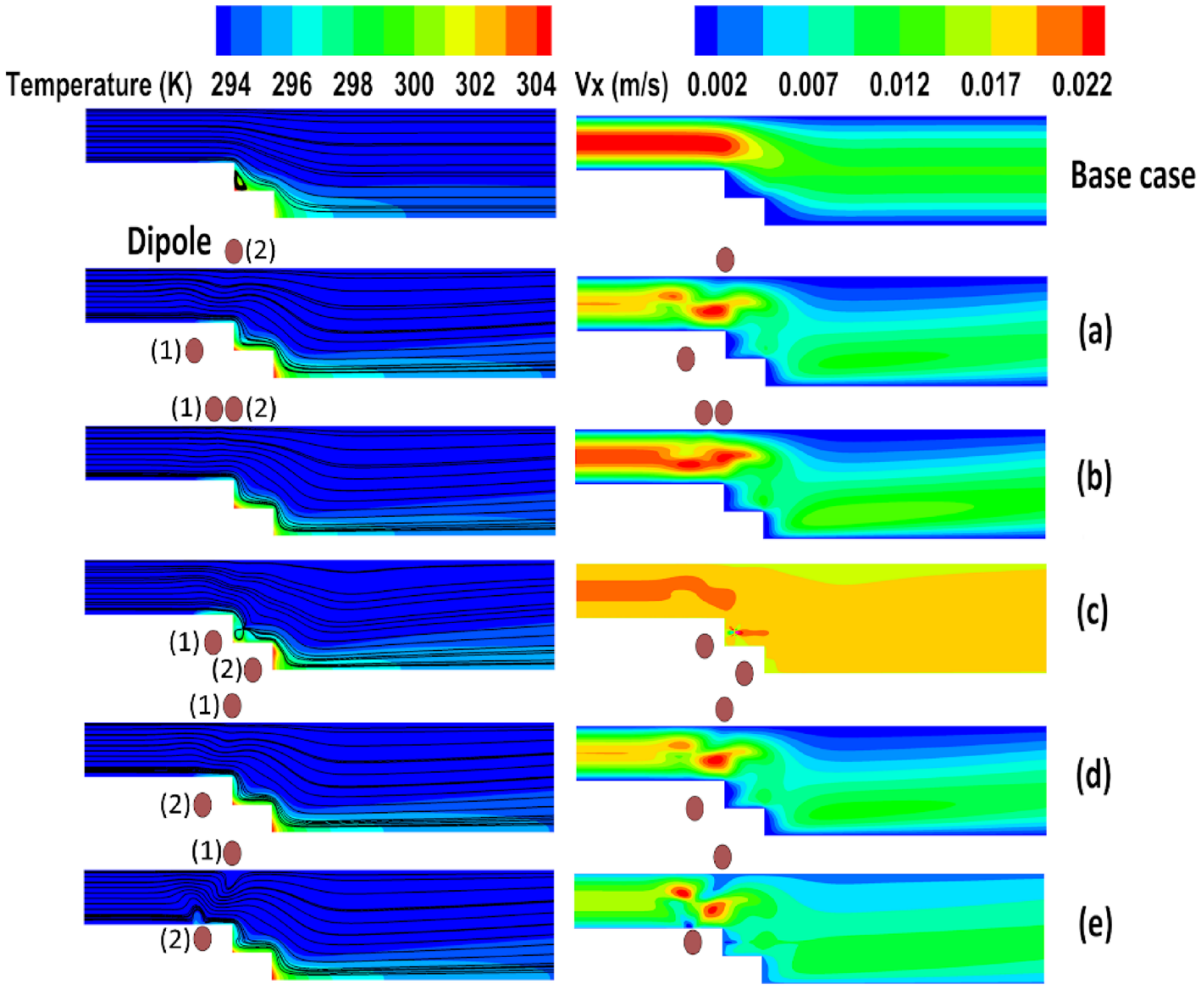
The contours of temperature and velocity and streamlines for different arrangements of double dipoles in the domain.

**Figure 15:**
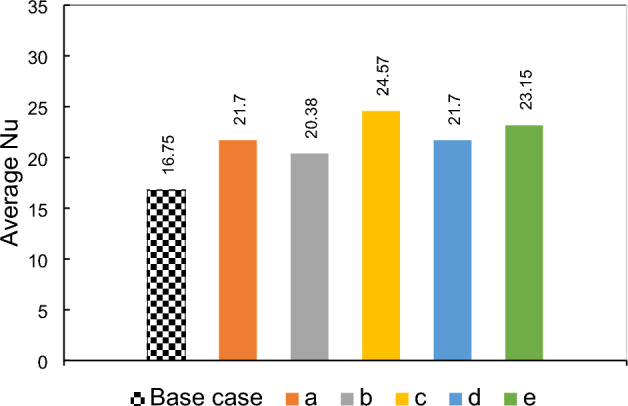
The average Nusselt number variation in different arrangements of double magnetic field in the domain.

In the preceding sections, the potential of employing a single dipole within the domain was assessed. Now, the investigation shifts focus towards examining the effects of utilizing two dipoles simultaneously in the domain. Each dipole can be positioned on either the upper or lower walls, resulting in various configurations. The geometrical parameters associated with dipoles refer to Fig. 1, depicted with subscripts 1 and 2, illustrating one of the dipoles. These dipoles are of equal strength; hence the sequence of the dipoles is insignificant. The local Nusselt numbers for different cases of the current investigation are presented in Fig. 13, highlighting significant changes in the flow for certain arrangements, such as configurations c and e. To elucidate these unexpected observations, Fig. 14 provides contours of flow velocity and temperature and streamlines. Analyzing this figure allows us to comprehend that in these extreme cases, the flow experiences localized wakes that abruptly disrupt the flow pattern. Consequently, within certain regions of these wakes, heat transfer is enhanced due to increased local convective heat transfer. Conversely, in other regions, the flow direction becomes opposite to the main flow, resulting in weakened heat transfer. For arrangements other than configurations e and c, the pre-existing wakes present in the case without a magnetic field are either weakened or amplified. Depending on the specific conditions, heat transfer may be enhanced or deteriorated accordingly. The flow behavior in this system is influenced by intricate interactions among variables such as pressure gradient, viscous forces, and the volumetric forces induced by the magnetic field. As a result, making precise predictions of the thermal behavior for other cases becomes challenging. However, the average Nusselt number can serve as a useful metric for identifying optimal arrangements. Figure 15 displays the average Nusselt numbers for different arrangements. It is evident that cases e and c exhibit higher average Nusselt numbers compared to the other cases. However, these arrangements may not be practical as they result in locally weak heat transfer on the first or second steps. Among the remaining arrangements, cases c and e demonstrate promising performance, exhibiting approximately 40% higher average Nusselt numbers than the base case. On the other hand, case b displays a lower average Nusselt number compared to the other arrangements. Therefore, while considering practical considerations, cases a and d emerge as favorable choices due to their improved performance in heat transfer. It should be noted that there is a fluctuation in the case e where the magnetic sources are placed before the expansion under the horizontal wall. Here the Kelvin forces impose a local recirculating zone before the step. This outcome is consistent with the previous research (for example the work of Bezaatpour and Goharkhah^46^). While in other cases the Kelvin forces synergize or weaken the main wake zone, appeared by the sudden expansion, here the wake zone operates independently. So, a fluctuation could be observed in the local heat transfer before the step.

### The influences of inlet Reynolds number

**Figure 16:**
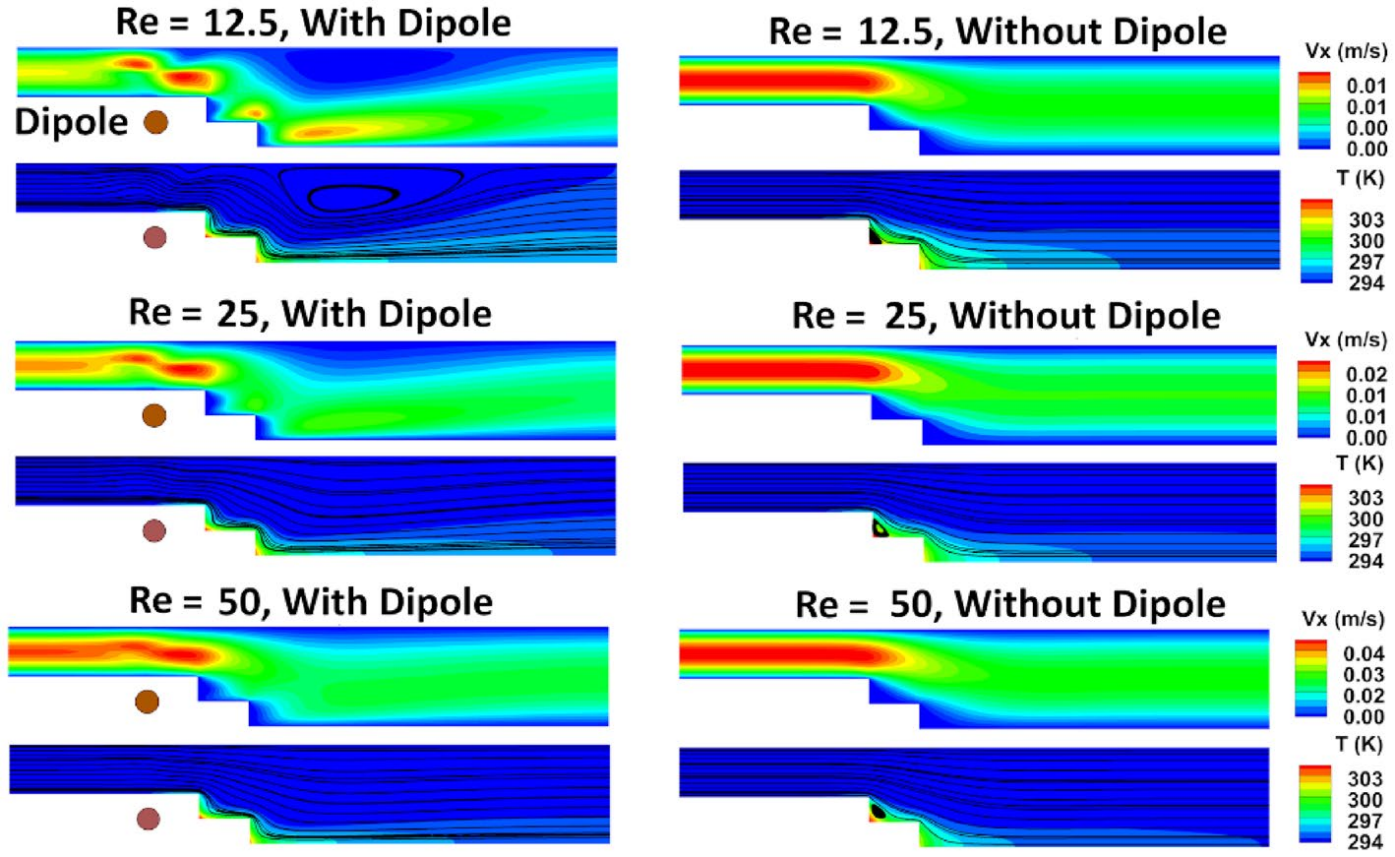
Flow patterns and contours for different Reynolds number (m = 1 A-m, Dipole is positiond under the lower border at a and b equal 19 mm and 0.5 mm, respectively, EMG-805 has used as inlet Fluid).

The flow Reynolds number reflects the ratio of inertia forces to viscous forces. As it increases, the inertia forces become more dominant, although it should be noted that the viscous forces may not remain constant, and their proportionality also increases. In essence, both forces rise as the Reynolds number rises. As the flow self-forces increase while the magnetic field parameters remain constant, the influence of the magnetic flow-induced forces becomes less significant. Therefore, as the Reynolds number decreases, the magnetic effects become more pronounced, and vice versa. Figure 16 presents the flow patterns and contours for different Reynolds numbers. It is evident that a large wake appears after the step. This is due to the magnetic forces accelerating the flow over the steps and enhancing the flow resistance against separation. After passing the steps, the flow seeks to recover itself by reducing velocity and increasing pressure. However, the adverse pressure gradient leads to undesirable separation and the formation of a wake. Nonetheless, this phenomenon occurs far from the steps, which are crucial from a heat transfer perspective. From the provided figure, it is evident that as the Reynolds number increases, the effect of the magnetic field diminishes.

**Figure 17:**
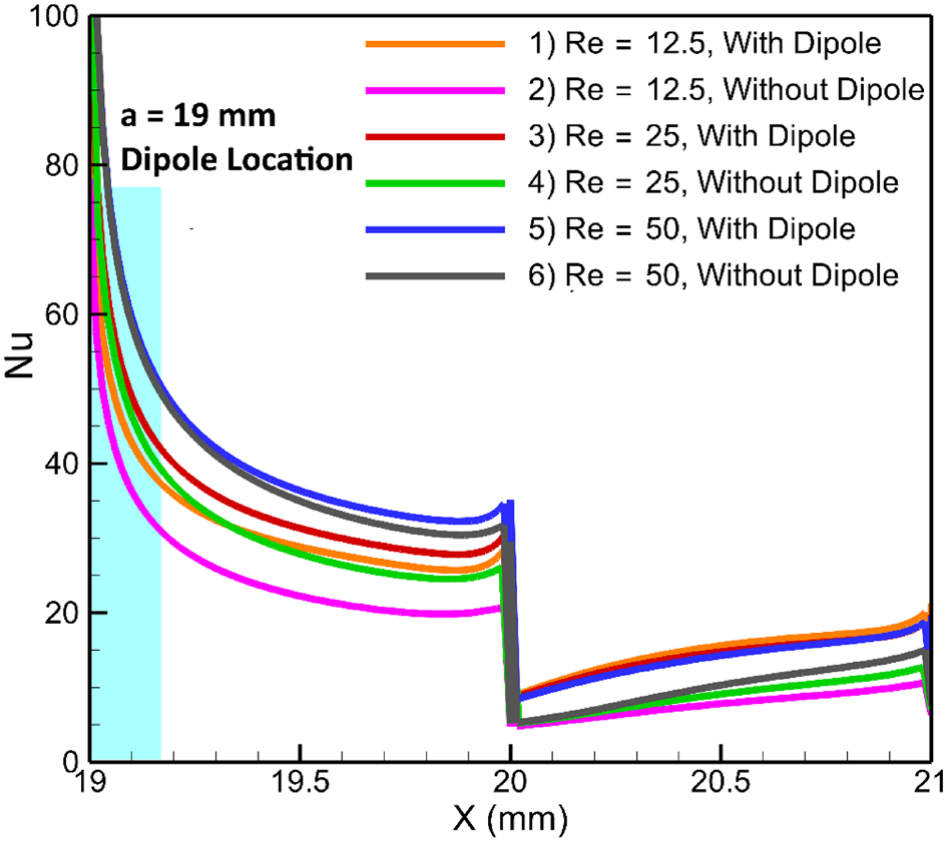
The variation of local Nusselt number against inlet flow Reynolds numbers.

**Figure 18:**
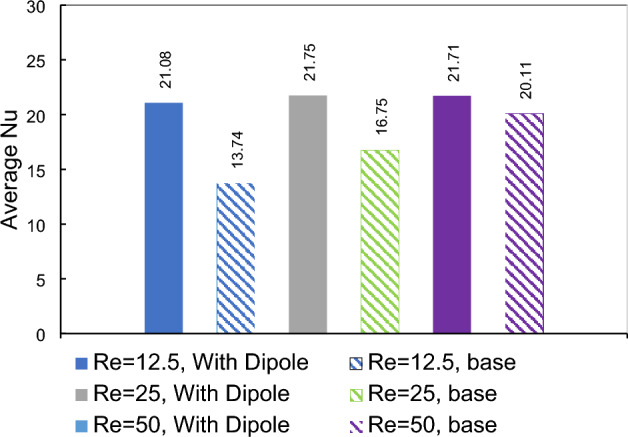
The variation average Nusselt number for several inlet flow Reynolds numbers.

Upon examining the local heat transfer depicted in Fig. 17, it becomes evident that the maximum enhancement cannot be easily discerned. However, by referring to the average Nusselt number represented in Fig. 18, a clearer picture emerges. This figure illustrates that the maximum overall thermal improvement is achieved at an inlet Reynolds number of 12.5, where the average Nusselt number increases by approximately 53%. This is followed by an improvement of about 30% at Re = 25 and 8% at Re = 50.

### Effects of dipole strength

**Figure 19:**
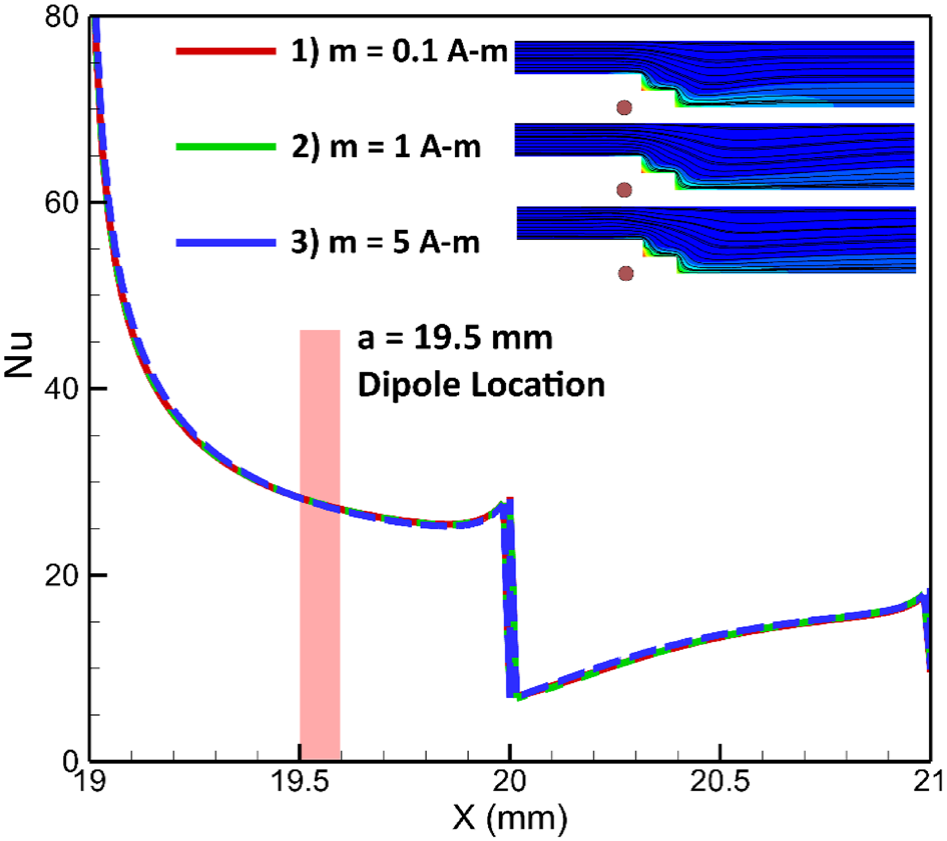
The dipole strength influences on the local heat transfer (Dipole is situated bellow the lower wall at a and be are 19.5 mm and 1 mm, respectively, EMG-805 has used as inlet Fluid, Re = 25).

**Figure 20:**
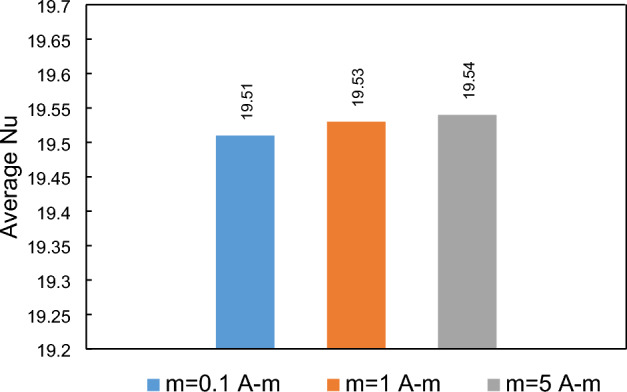
The impacts of dipole strength (located bellow the lower border) on the average Nusselt number.

The strength of the dipole is another significant parameter that influences the flow patterns^47–49^. This variable directly affects the volumetric force emerged by the magnetic source. In this analysis, two arrangements are examined: cases where the dipole is positioned on the upper or lower wall. Figure 19 displays the influences of the strength of dipole on the local heat transfer when the dipole is located under the lower border. It can be concluded that, under these conditions, the local Nusselt number is not significantly affected by the strength of the dipole located at the specific location. This observation is further supported by Fig. 20, which presents the average Nusselt number. Hence, both figures indicate that the dipole strength, when fixed at a specified location, does not have a considerable impact on the average and local Nusselt numbers.

**Figure 21:**
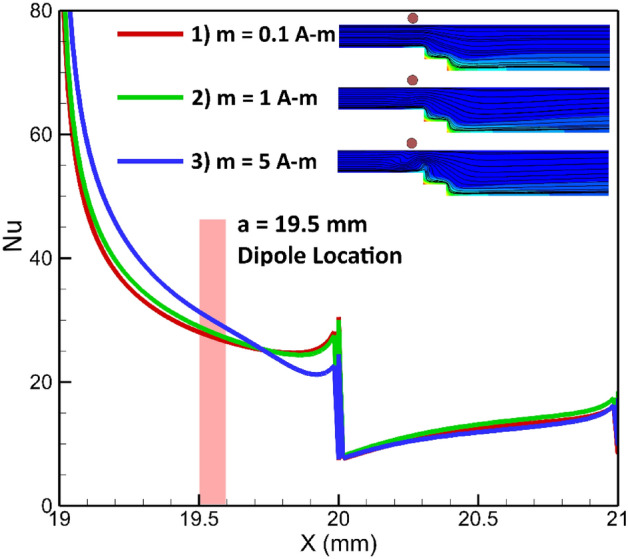
The impacts of magnetic source strength on the local heat transfer (Dipole located over the upper wall at a and be are 19.5 mm 0.5 mm, respectively, EMG-805 has used as inlet Fluid, Re = 25).

Figure 21 shows when the dipole locates on the upper wall at a fixed position, the outcomes differ somehow. It can be seen that when the dipole strength inceases, the local Nusselt number rises considerably in the first part (19 < a < 20 mm). the main reason is pushed by the magnetic force on the first section.

**Figure 22:**
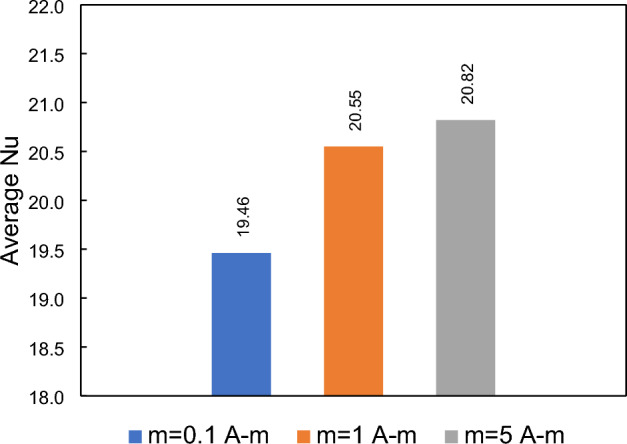
The impacts of dipole strength (located over the upper border) on the average Nusselt number.

The average Nusselt number for different strengths of the magnetic source, located over the upper border, is presented in Fig. 22. It is obvious that increasing the strength of the dipole leads to improved heat transfer. For instance, increasing the dipole strength by 50 times results in approximately a 24% enhancement in the average Nusselt number compare to the base case. The relatively small increase in the Nusselt number could be attributed to the remote location of the dipole with respect to the step location. Since the dipole is not placed directly near the steps, its influence on heat transfer may be somewhat limited. Nonetheless, the results demonstrate that increasing the strength of the dipole can positively impact heat transfer, albeit to a moderate extent.

## Conclusion

In conclusion it can be said that while previous numerical and experimental studies have explored various techniques to enhance heat transfer in channels with backward-facing steps, but gaps remain. Ferrofluids and magnetic fields in double backward-facing steps are underexplored despite their potential for improved heat transfer. Factors like magnetic dipole number and strength, Reynolds number, and magnetic source location require further investigation to optimize thermal management. This study investigates magnetic dipoles' impact on heat transfer and fluid flow in a millichannel with consecutive expansions, considering various factors. Key findings include:

- The type of ferrofluid used had a significant impact on the cooling rate, with a higher volume fraction of magnetic particles in the base fluid resulting in a higher cooling rate.
- Adjusting the longitudinal position of a magnetic dipole on the lower wall led to a 22% improvement in the average Nusselt number with respect to the case without a magnetic field.
- Placing a magnetic dipole closer to the channel wall, underneath the lower border, resulted in a 12% improvement in the average Nusselt number with respect to other locations of the magnetic field.
- Similar to the lower wall, adjusting the longitudinal position of a magnetic dipole on the upper border increased the average Nusselt number by 22% with respect to the case without a magnetic field.
- By strategically placing two magnetic dipoles in optimal locations, the cooling rate and average Nusselt number increased by approximately 40% with respect to the conditions without a magnetic field.
- Varying the Re had an influence on the impact of the volume force caused by the magnetic dipole. Rising the Reynolds number reduced the influences of the magnetic force due to the increased inertial force of the fluid in the conduit.
- Changing the strength of the magnetic dipole resulted in an increase in the cooling rate. However, the magnitude of this increase depended on the location of the magnetic dipole. Placing the magnetic dipole on the upper wall and increasing its strength led to a greater improvement in the average Nusselt number with respect to placing it on the lower wall.

### Future directions

- Employing hybrid nanofluids, combining highly conductive nanoparticles like copper with magnetic particles, to enhance fluid conductivity and control flow dynamics.
- Conducting extensive simulations considering simultaneous or separate parameter effects. Employing algorithms such as artificial neural networks to establish correlations between inputs and outputs.
- Utilizing optimization algorithms to identify optimal points based on established correlations.
- Investigating the effects of various ferrofluid compositions on the problem.

## List of symbols

a, b: Coordinate (m)
B: Magnetic flux (T)
C_p_: Specific heat (kJ/kg.^o^C)
D_h_: Hydraulic diameter (m)
e_r_, e_θ_: Unit vectors
H: Magnetic field strength (A/m)
H_1_, H_2_, H_s_: Parameters in Fig. 1 (m)
h: Heat transfer coefficient (W/ (m^2^.K))
k: Thermal conductivity (W/m.K)
K_B_: Boltzmann constant (J/K)
L_1_, L_2_, L_s_: Parameters in Fig. 1 (m)
M: Magnetization (A/m)
m: Magnetic dipole strength (A-m)
Nu: Nusselt number
P: Pressure (Pa)
q″: Heat flux (W/m^2^)
Re: Reynolds number
r, θ: Polar coordinate
S_x_, S_y_: Source terms (N/m^3^)
u, v: Velocities (m/s)
X, Y: Cartesian coordinate
T: Temperature (^o^C, K)
ρ: Density (kg/m^3^)

## Subscripts

d: Domain
s: Saturation
in: Inlet
